# Unidirectional growth of organic single crystals of naphthalene, anthracene and pyrene by isothermal expansion of supercritical CO_2_†

**DOI:** 10.1039/d0ra03706k

**Published:** 2020-06-11

**Authors:** Antaram Sarve, Jimil George, Santosh Agrawal, Raksh Vir Jasra, Pradip Munshi

**Affiliations:** Department of Chemical Engineering, Sardar Vallabhbhai National Institute of Technology Surat 395007 India; Department of Chemistry, Cochin University of Science and Technology Cochin Kerala 682022 India; Research Centre, Reliance Technology Group, Reliance Industries Limited Vadodara Gujarat 391346 India pradip.munshi@ril.com

## Abstract

Unidirectional single crystals without grain boundaries are highly important in optoelectronic applications. Conventional methods to obtain such crystals involve organic solvents or seed crystals, which have numerous drawbacks. We present here a supercritical CO_2_-mediated method of the single crystal formation of naphthalene, anthracene and pyrene on the (001) plane without using seed crystals. Single dominant peaks in powder XRD (PXRD) with low full width at half maxima (FWHM) are described. The dependency of crystal size on the rate of depressurization was measured by precise and isothermal expansion of scCO_2_ solutions. The experimental setup is illustrated for continuous preparation without emission of CO_2_ or discharge of material into the environment. The materials are shown to be fully converted into crystals indicating a rapid, scalable and environmentally benign process of single crystal formation with practically nil E factor.

## Abstract 2

Slow isothermal expansion of a supercritical CO_2_ solution resulting in unidirectional single crystals of controllable size as a method of crystallization with practically nil E factor.

## Graphical abstract

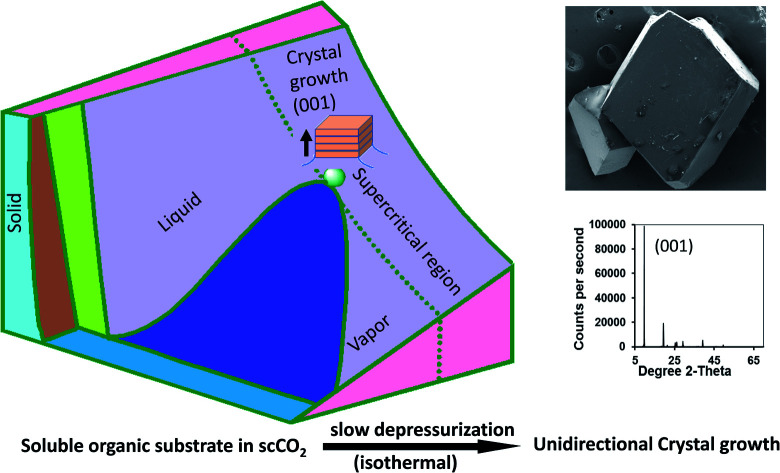

## 1. Introduction

**Fig. 1:**
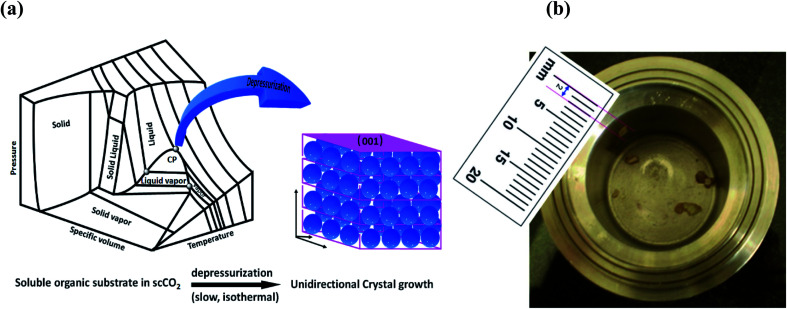
(a) Schematic representation of unidirectional crystal formation in the supercritical region. (b) Anthracene crystal formed inside the vessel.

Unknown to date, the masoning of molecules is demonstrated by growing organic crystals (naphthalene, anthracene and pyrene) unidirectionally on the (001) plane through isothermal expansion of a supercritical CO_2_ (scCO_2_) solution (Fig. 1a). Unidirectional single crystals^1^ without grain boundaries have huge potential and are highly important in optoelectronic applications,^2^ and of course are highly demanded in the preparation of advanced materials, namely for data storage, communication high energy lasers and electro-optic switches.^3^ Uniform deposition under the unit face with minimum grain boundaries and maximum gain in Gibbs free energy is a prerequisite for getting a perfect crystal.^4^

The ability to grow unidirectional large single crystals is one of the essential criteria to satisfy the needs of science and technology.^1,5^ In academia, single crystals are unavoidably considered as one of the fundamental requirements^6^ to reveal the absolute molecular arrangements.^7^ However, the solvent diffusion and vapor deposition methodologies that are commonly used have genuine drawbacks, like solvent usage, solvent inclusion, uncertainty in getting perfect crystals, grain boundaries *etc.*^8^ Crystallization is a thermodynamic preference of solute molecules from solution in which viscosity (*η*) and surface tension (*γ*) play vital roles. These properties are almost unchangeable for organic solvents and have very stringent requirements to generate the requisite thermodynamic conditions for crystallization. On the other hand, for supercritical fluids (SCFs) these properties are tunable and can achieve the necessary supersaturation to facilitate the process of primary nucleation, which is essential for crystallization.^9^

SCFs have numerous applications in material processing and crystallization due to their unique properties, namely high diffusivity (diffusion coefficient 70 000 m^2^ s^−1^), high compressibility and higher solvency.^10^ It was perhaps Hannay and Hogarth who first observed particle formation from a supercritical solution in 1879.^11^ They described the phenomenon as ‘formation of snow’. However, this pioneering finding was unnoticed for about 100 years until Krukonis in 1985 took an interest and investigated the economic viability of supercritical fluid-mediated processes.^12^ Smith and coworkers later referred to the process as ‘rapid expansion of supercritical solutions’ (RESS).^13^

Post the discovery of RESS, many reports cite crystallization through a SCF. Supercritical CO_2_ (scCO_2_) has additional advantages because it is a green solvent^14–16^ and has an easily attainable critical temperature (*T*_c_ 31.0 °C) and pressure (*P*_c_ 73.8 bar), primarily for smaller or nanoparticle generation.^17^ Gas anti-solvent (GAS) or solution anti-solvent (SAS) are other techniques commonly used to generate small particles from SCFs. These methods have been progressively extended to polymer nucleation as well.^18^ Several theories have been put forward for proper modelling of particle formation in scCO_2_.^19,20^ However, there are only a few reports in the literature regarding single crystal formation in scCO_2_, as detailed below.

Slow expansion of supercritical CO_2_ solution (SESS) was carried out by Tai and Cheng^21,22^ in 1995, and they obtained 2 mm naphthalene crystals in the presence of a seed crystal. Not much detail about the nature of the crystal was disclosed. Later, Raveendran *et al.*^23^ in 2005 prepared single crystal of a carbohydrate solution in CO_2_ through SESS. About a decade later, Pessi *et al.* revealed a subsidiary technique named controlled expansion of supercritical solution (CESS), which primarily provides larger nanoparticles with narrow particle distribution at a slow rate of expansion.^24^ The hydrothermal route of single crystal formation is very popular for inorganic and metal–organic framework (MOF)-type materials, and occurs mainly in super or sub-critical water, but no organic molecules have been reported so far, perhaps due to high temperature.^25^

It is worth mentioning a few literature reports that are loosely relevant to CO_2_-mediated single crystal formation. MgCO_3_ from a reaction of molten sodium with CO_2_ at 550 °C,^26^ and Fe-ferrocene^27^ are a couple of available examples of single crystal formation in scCO_2_. Diamond is reported to be recrystallized in CO_2_ by *in situ* reduction of CO_2_.^28^ Uchida^29^ obtained naphthalene crystals in CO_2_ in the presence of a seed crystal, attaining supersaturation by varying the concentration and temperature. However, the above literature reports lack precise control of the temperature and rate of expansion, and that is what inspired the present study.

We have observed that isothermal, slow and precise expansion of supercritical CO_2_ solutions of naphthalene, anthracene and pyrene at mild temperatures can generate unidirectional single crystals selectively on the (001) plane (Fig. 1a and b), without introducing any seed crystals, which was not revealed earlier. Such conditions of supercritical fluids provide a better environment to attain large-size unidirectional crystal formation, as shown in Fig. 1a in the three dimensional phase diagram of CO_2_.^30^ In fact, this method is direct, rapid and environmentally benign, energy efficient, and holds promise for continuous production recycling the CO_2_ with practically nil E factor, which is the ratio of waste to product mass.^31^ This has huge potential in the organic scintillator market, which is expected to reach 480 million USD by 2020 with a 5.6% compound annual growth rate (CAGR).^32^ Other related potential market areas include organic semiconductor materials and field effect transistors (FETs).^33^

## 2. Experimental

### 2.1. Materials and characterization

The high pressure reactor was purchased from Parr Instrument company, USA. The gas booster, Haskel Model AG 160, was purchased from Haskel, Accudyne Industries Brand, USA. XRD was performed on a Bruker, Model D8 ADVANCE, with Cu K-α beam radiation. The generator was operated at 40 kV and 40 mA, with step size = 0.02° 2*θ* and a counting time of 5 seconds, and the data were analyzed through Defrac.Suite EVA software. Diffracted X-rays were recorded on a scintillation counter detector. SEM was performed on an FEI Ltd Model Nova NanoSEM 450. The flowmeter was purchased from Cole-Parmer, USA. The vernier scale used to measure the crystal length was a Mitutoyo, Model CD 12" PSX. CO_2_ and air cylinders were purchased from Ultra Pure Gases (I) Pvt. Ltd. India. The full width at half maximum (FWHM) was calculated using Origin Pro 9.1 64-bit software.

Naphthalene, anthracene and phenanthrene were purchased from Sigma-Aldrich Inc. Anal. Grade and used without further purification. Compressed CO_2_ was purchased from Praxair India, with >99.5% purity.

### 2.2. Preparation of crystals through slow expansion of a supercritical CO_2_ solution

**Fig. 2:**
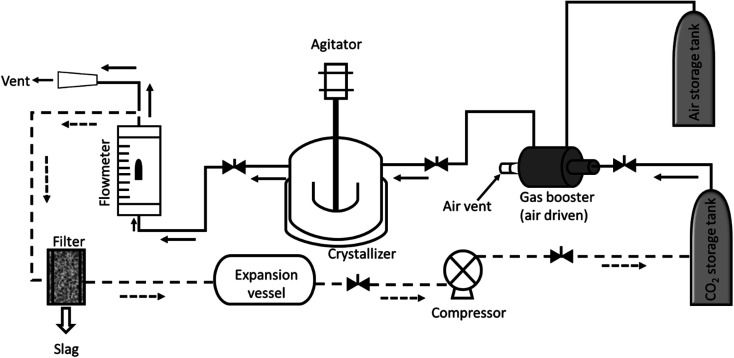
Experimental set up used for crystallization. The crystallizer is jacketed and connected to a polyscience water chiller to maintain a constant temperature. Dotted line ( <svg xmlns="http://www.w3.org/2000/svg" version="1.0" width="37.000000pt" height="16.000000pt" viewBox="0 0 37.000000 16.000000" preserveAspectRatio="xMidYMid meet"><metadata> Created by potrace 1.16, written by Peter Selinger 2001-2019 </metadata><g transform="translate(1.000000,15.000000) scale(0.014583,-0.014583)" fill="currentColor" stroke="none"><path d="M80 440 l0 -40 320 0 320 0 0 40 0 40 -320 0 -320 0 0 -40z M880 440 l0 -40 320 0 320 0 0 40 0 40 -320 0 -320 0 0 -40z M1680 440 l0 -40 320 0 320 0 0 40 0 40 -320 0 -320 0 0 -40z"/></g></svg> ) represents a proposed set up for continuous preparation.

In a typical experiment, 2 g of anthracene was taken in a 100 mL Parr reactor and gently flashed with air and CO_2_. An air driven gas booster was connected with the cylinder and switched on, and CO_2_ was pumped into the reactor. The desired pressure was obtained with accuracy ±2 bar. The temperature of the reactor was maintained at 45 °C ± 2 °C. Stirring was started and continued for 1 h. The outlet of the vessel was connected to the flowmeter with 10 mm tubing. The vessel was left for 1 h to equilibrate the solution at the set temperature. Slow depressurization was started by opening the valve at a constant temperature. The gas was measured with respect to time as collected though a pneumatic trough (Fig. S1†). After complete depressurization, the vessel was carefully opened and the crystals collected. The crystals were collected for characterization. A schematic drawing of the experimental set up is shown in Fig. 2.

Some powder was left, which could not be converted into crystals. As a result, the crystals were separated from the powders manually, as much as possible, and weighed. The yield calculation was done based on the crystals obtained against initial weight of material taken. The yield of the crystals calculated as percent mass transformation is shown in Table 1. Other substrates (naphthalene and pyrene) are represented in a similar fashion.

**Table 1:** Results of crystallization from CO_2_ solutions of different substrates^a^

Sample	XRD intensity (001)^b^ [counts per second]	Crystal length^c^ [mm]	FWHM^d^	Depressurization rate [mL per min]	% Yield^e^ [(*W*_c_/W_0_) × 100]
Naphthalene	16 881	3.00	0.0856	10	67.09
	7256	1.66	1.2512	20	
Anthracene	100 000	2.00	0.0689	10	45.8
	2800	1.37	0.3275	20	
	130	0.12	—	50	
Pyrene	15 250	3.33	0.0823	10	32.7

a CO_2_ 120 bar, temp. 45 °C.

b XRD intensity considered for the peak corresponding to the (001) face.

c Length measured using a Vernier scale (Mitutoyo), with average error ±0.5.

d FWHM determined using Origin Pro 9.1.

e *W*_0_ initial weight, *W*_c_ weight of crystal. Naphthalene (*W*_0_ 2.012 g, *W*_c_ 1.35 g); anthracene (*W*_0_ 2.008 g, *W*_c_ 0.92 g); pyrene (*W*_0_ 2.017 g, *W*_c_ 0.66 g).

A similar experiment was done without taking the organic substrate and monitoring the temperature and pressure. It was observed that there is no observable change in temperature during depressurization. This indicates that cooling or temperature fluctuations due to depressurization do not significantly affect crystal formation, or it could be that the minute heat fluctuations were eliminated by the insulation jacket equipped to the crystallizer vessel.

For a continuous process, the experimental set up is proposed as described in Fig. 2 (dotted line). The vent of the flow meter could be connected to the expansion vessel *via* a filter or separator to remove the particulates, if any. The expanded gas could be compressed through a compressor and used back in the cycle.

To utilize all of the material in crystal formation, the powder left was used in the next experiment with an additional amount of the same substrate. The experiment continued in a similar fashion, as described.

## 3. Results

**Fig. 3:**
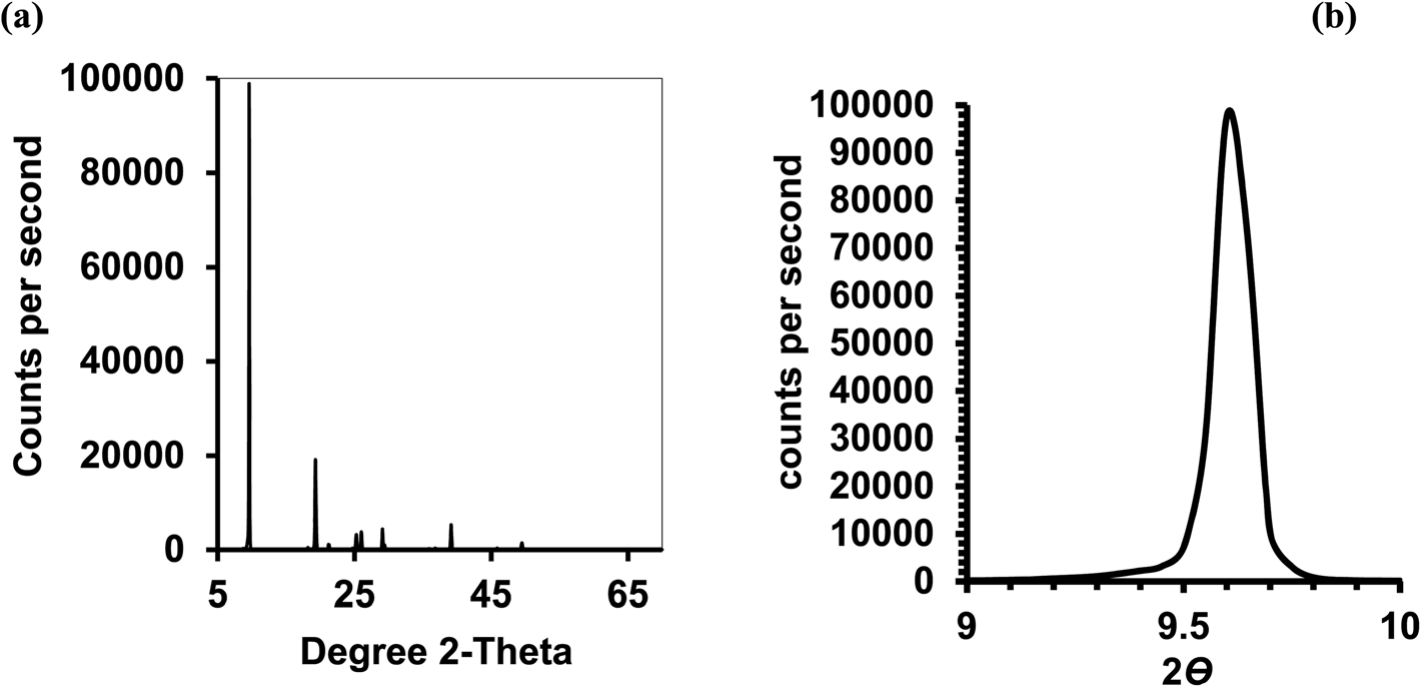
Anthracene crystal; CO_2_ 120 bar, temp. 45 °C, depressurization 10 mL per min. (a) Powder XRD of (b) the rocking curve.

**Fig. 4:**
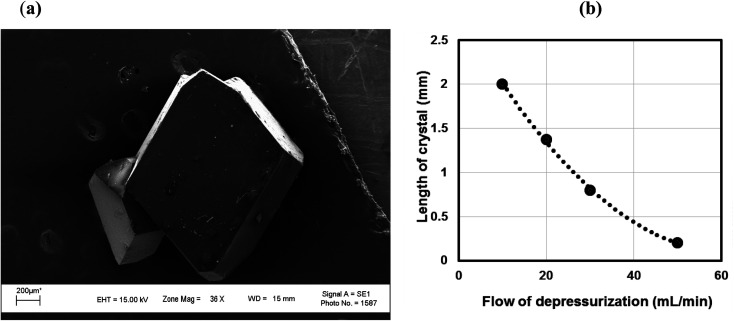
(a) SEM picture of an anthracene crystal. CO_2_ 120 bar, temp. 45 °C, depressurization 10 mL per min. (b) Length of the anthracene crystal monitored with respect to rate of depressurization. CO_2_ 120 bar, temp. 45 °C.

**Fig. 5:**
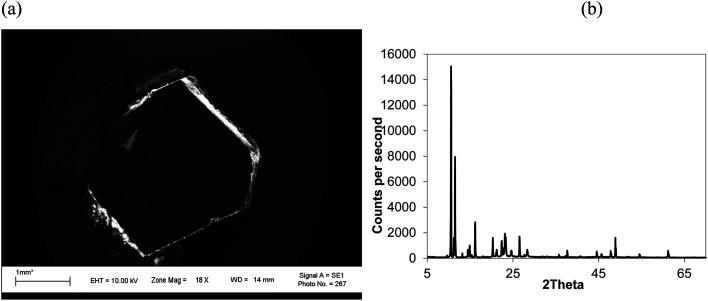
(a) SEM image and (b) XRD of a pyrene crystal. CO_2_ 120 bar, temp. 45 °C, depressurization 10 mL per min, FWHM for 2*θ* 10.55° (001) 0.0823.

Isothermal depressurization of supercritical CO_2_ solutions (namely naphthalene, anthracene, pyrene) yields large size single crystals up to 3.33 mm in length unidirectionally on the (001) plane (Table 1). After depressurization, good shiny crystals were obtained, as shown in Fig. 2b. Powder XRD of the anthracene crystal after depressurization shows (Fig. 3a) a very high intensity (100 000 counts per second) majorly dominating peak at 2*θ* of 9.5°, corresponding to the (001) plane of anthracene,^34^ which is evidence of a high degree of crystallinity and unidirectional growth along the (001) plane.^35^ The FWHM of this peak determined from the rocking curve (reveals broadening of the XRD peak) is 0.0689 (Fig. 3b), indicating that the crystals have less dislocation densities, minimum grain boundaries,^33,35^ larger size and higher strength.^36^ The SEM image of the anthracene crystal shown in Fig. 4a depicts the cuboid shape of the crystal with sharp edges. Fig. 4b displays a representative plot of anthracene crystal size *versus* depressurization rate. In a similar manner, naphthalene and pyrene crystals were obtained with a length of 3 mm and 3.33 mm, respectively (Fig. S2a† and Fig. 5a, respectively). The singly dominating intense XRD peak of naphthalene observed at 2*θ* 13.95° (Fig. S2b†) matches with the literature value of 14.0°,^37^ and that of pyrene observed at 2*θ* 10.6° (Fig. 5b) matches with the literature value of 10.6°,^38^ designated as (001) plane reflections. The FWHM values of these crystals are 0.0856 and 0.0823, respectively.

**Fig. 6:**
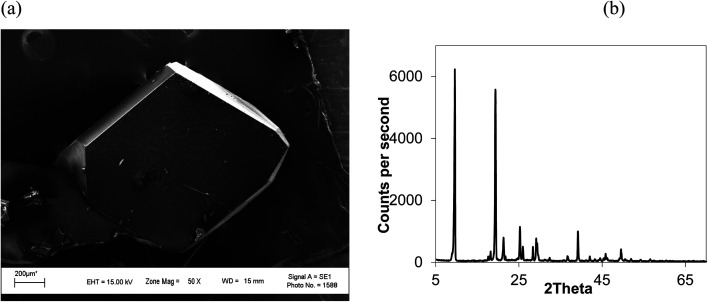
(a) SEM image and (b) XRD of an anthracene crystal at higher depressurization (20 mL per min). CO_2_ 120 bar, temp. 45 °C, FWHM for 2*θ* 9.6° (001) 0.3275.

Unlike rapid expansion of supercritical solution (RESS), the particle size of the crystal is inversely proportional to the rate of expansion pursued. Depressurization at 10 mL per min provides crystals of 2 mm length and at a depressurization rate of ∼50 mL per min, the crystal size becomes 0.12 mm (Table 1, Fig. S3†). The size of the crystals is inversely related with the rate of depressurization. Not only the size, but also the quality of the crystals become inferior at higher depressurization, as depicted in the SEM image (Fig. 6a) with grain overgrowth and XRD (Fig. 6b) with lower intensity and multiple faces, and also the high FWHM of 0.3275 when an anthracene crystal was obtained with a depressurization rate of 20 mL per min.

At higher temperature, the crystallization rate is faster but the crystal size becomes smaller and a loss in crystallinity is also observed. This is attributed to the metastable zone width being narrower at higher temperature and the probability of getting a single crystal decreasing.^39^ Unconverted material was reused in the next stage of crystal formation. The results (Table S1†) show that the material loss is practically negligible.

## 4. Discussion

There are a few literature reports cited below that could provide some insight into this phenomenon. The classical nucleation theory (CNT)^40^ derived for solid-solute solution from CO_2_ by Liang and Wang dictates that the crystal nucleus density (*N*_cell_) is a function of the rate of depressurization (d*P*/d*t*) and the depressurization of a solid solute–CO_2_ solution tends to form nuclei at a higher rate than that from a homogeneous supersaturated solution,^41^ where eqn (1) represents the energy barrier during depressurization. Debenedetti theorized the homogeneous nucleation rate of phenanthrene–CO_2_ solution at different depressurization conditions (isothermal and isobaric) of RESS (rapid expansion of supercritical solution) using CNTs and revealed that for isothermal conditions, the attainable nucleation rate is steady.^42,43^ Goel and Beckman^44^ applied CNTs over poly(methylmethacrylate) (PMMA) foam in CO_2_ and stated that the size of the critical nucleus is largely dependent upon the pressure. Liang and Wang^41^ found two-stage depressurization of PET foam from CO_2_ solution to be more effective at providing higher nucleus densities than one-stage depressurization.

In the present case, the anthracene solution was very slowly depressurized from 120 bar until atmospheric pressure, which might have allowed the crystals to grow bigger. The particle size observed was less when the depressurization rate was higher, and also the particle size was less when depressurization was done at higher pressure. This is in agreement with the literature, as particle size decreases with an increase in post-depressurization pressure.^43^ Tai and Cheng observed that during the crystallization of naphthalene from CO_2_, pressure fluctuations provided much lower supersaturation than temperature disturbances, and higher supersaturation and lower velocity rendered the crystal size smaller.^21^ At a pressure of 120 bar, and temperature of 45 °C, pyrene crystals with a length of 3.33 mm, naphthalene crystals with a length of 3.00 mm and anthracene crystals with a length of 2.00 mm were obtained (Table 1). Comparatively, the perfectness of the crystals was seen to be higher for anthracene as depicted in the SEM image, XRD intensity and FWHM, than for naphthalene, followed by pyrene.

Burton, Cabrera and Frank^45,46^ proposed a screw dislocation theory, known as BCF (Burton, Cabrera and Frank) theory, which seems to be more satisfactory in the present case. The theory explains the growth of crystals at low supersaturation by diffusion of atoms over the surface of the primary crystal and remaining on top of the step formed by the two planes. The crystal surface traverses through a helical ramp arranged in the direction of a right- or left-handed screw. The nucleation at the edge dislocation is influenced by the surface stress, which provides the extra energy for formation of primary nuclei. The free energy of nucleation is highly favorable near the critical point, while the crystallization begins with the formation of a dense liquid droplet that induces primary nucleation.^47^ Depressurization of CO_2_ triggers new surface formation facilitating primary nucleation. Thus, slow depressurization allows the crystal to form uniformly and grow in a facile manner on the (001) plane, and might provide a suitable condition for Wulff construction.^48^ Chevalier's recent discovery is even more insightful at this stage, which revealed that surface tension at the gas–liquid interface is the responsible factor for orientation of crystals.^49^ The rate of orientation is obtained by the equation *τ* = *ηa*/*γ* (*τ* is the torque governed due to capillary force of orientation building up, *η* is the dynamic viscosity of the liquid, *a* is the crystal size and *γ* is the surface tension). This is the situation perhaps achieved by depressurization of scCO_2_ making the crystal growth unidirectional, which the bulk crystallization techniques are not able to provide. Li *et al.* explained vapor-transport growth of two-dimensional (2D) hexagonal boron nitride (h-BN)^50^ nanoplates and niobium diselenide (NbSe_2_)^51^ using anthracene as a single-molecule intercalant due to its perfect 2D structure having low vapor pressure.

A spectacular observation by Balamurugan^52^ noted that bulk crystallization of naphthalene ended up with many faces, but Postnikov^37^ obtained primarily (001) faced crystals during crystallization of naphthalene from an air–liquid interface.

Solution borne crystallization takes several days to form the crystal, whereas the present method can execute crystal formation in a very short time. Moreover, the unutilized material was recycled in the next stage of crystal formation with the added amount of substrate (Table S1†) keeping the yield the same. Theoretically, the material can be converted completely into crystals in three cycles of crystallization. Though CO_2_ after depressurization could not be recycled (due to the unavailability of a compressor), recycling CO_2_ after compression is a well-known art.^53^ A diagram is proposed describing the process of crystallization (Fig. 2, dotted line). So unambiguously, the system can be set-up for semi-continuous production of single crystals, effectively with no discharge to the environment or a nil E factor.1
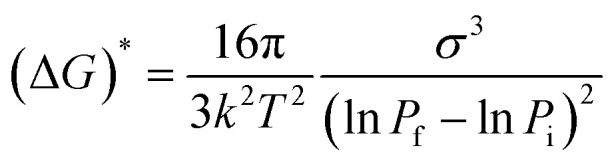


Table 2 shows the crystallization of some of these substrates as a comparison with references and the present work. The data is self-explanatory; crystals are obtained with a single face by slow depressurization of CO_2_. We believe that the scCO_2_ mediated method could provide commercial opportunity for a next generation environmentally benign continuous process for the preparation of unidirectional crystals.^54,55^ This would have huge societal impact in terms of its cost and availability as the modern era is foreseeing high demand of such organic crystals in the forthcoming advanced digital gadgets.^1^

**Table 2:** Comparison of crystallization of substrates with relevant reference work

Substrate	Length (mm)	Seed crystal	CO_2_ (bar)	Growth rate^a^ (m s^−1^)	Unidirectional (001)	Expansion technique	Reference
Naphthalene	0.40	Yes	78	4 × 10^−7^	No (multifaced)	Slow	21 and 22
Naphthalene	0.20	Y	221	23	No (multifaced)	Rapid	42
Naphthalene	0.3	Y	150	2.1 × 10^−7^	Yes (multifaced)	Controlled by T, P	29
Anthracene	2.20	No	—	9.1 × 10^−9^	No (multifaced)	Solution	34
Anthracene	10	No	—	1.15 × 10^−9^	Yes (001)	Air–liquid interface	37
Anthracene	2.00	No	120	2.8 × 10^−8^	Yes (001)	Slow, precise, isothermal	Present work
Naphthalene	3.00	No	120	4.22 × 10^−8^	Yes (001)	–do–	–do–
Pyrene	3.33	No	12	4.69 × 10^−8^	Yes (001)	–do–	–do–

a ESI for calculation of growth rate.

## 5. Conclusion

In conclusion, unidirectional single crystals were obtained by precise and isothermal expansion of scCO_2_ solutions of naphthalene, anthracene and pyrene without using any seed crystals. Controllable size up to 3.33 mm of single crystals grown unidirectionally on the (001) plane was observed, with a crystal efficiency up to 70%. A single dominating XRD peak with an intensity of 1 × 10^5^ counts per s was observed. The growth rate obtained was on the order of 10^−8^ m s^−1^, ∼10 times faster than solvent-borne crystallization. The size of the crystal is inversely proportional to the rate of depressurization. This method provides complete material utilization and CO_2_ recyclability, and could be considered as a next generation environmentally benign continuous process for the commercial preparation of large-size organic crystals.

## Conflicts of interest

There are no conflicts to declare.

## Supplementary Material

RA-010-D0RA03706K-s001
