# Supplementary material for: Unidirectional growth of organic single crystals of naphthalene, anthracene and pyrene by isothermal expansion of supercritical CO2
Source: RSC Adv. 2020 Jun 11;10(38):22480–6. doi: 10.1039/d0ra03706k (PMC9054696; doi:10.1039/d0ra03706k)
Supplement: RA-010-D0RA03706K-s001 [file RA-010-D0RA03706K-s001.pdf]

Electronic Supplementary Material (ESI) for RSC Advances.
This journal is © The Royal Society of Chemistry 2020

Supporting Information

Unidirectional growth of organic single crystals of naphthalene, anthracene and pyrene by isothermal expansion of supercritical CO_2

*Anant Sarve[‡], Jimil George[†], Santosh Agrawal[¶], Raksh Vir Jasra[¶] and Pradip Munshi[¶]**

[‡] Department of Chemical Engineering, Sardar Vallabhbhai National Institute of Technology, Surat, 395007, India.

[†]Department of Chemistry, Cochin University of Science and Technology, Cochin, Kerala 682022, India.

[¶]Research Centre, Reliance Technology Group, Reliance Industries Limited, Vadodara, Gujarat, India, 391346, India.

**Content**	**Page**
Index	1
Measurement of depressurized CO_2.	2
Determination of rate of crystallization	2
Table S1: Recycle test of anthracene crystallization.	3
Fig. S1. Pneumatic trough gas collection for measurement of depressurized CO_2 gas collected to crystallizer vent.	3
Fig S2. a) SEM Image, b) XRD of naphthalene crystal. CO_2 120 bar, temp. 45°C, depressurization 10 mL per min, FWHM for 2θ 13.96° [001] 0.0856.	4
Fig. S3. a) SEM Image, b) XRD of anthracene crystal. CO_2 120 bar, temp. 45°C, depressurization 50 mL per min.	4

1. Measurement of depressurized CO_2.

Depressurized CO_2 was measured through pneumatic trough as described in Fig S2. The outlet of crystallizer vent was connected to the inlet shown by "→". A flat container of 5 L (1) was taken and filled with 2.5 L water. A graduated cylinder (2) (diameter 20 cm, height 10 cm) was completely filled with water and placed inverted into 1. Incoming CO_2 from crystallizer vessel as shown through arrow (→) was passed through the tube (3) that is inserted under water into 2 as shown in Fig S2. Collected CO_2 (4) was measured with respect to time as per the pre-calibrated water level.

2. Determination of rate of crystallization

The rate of crystallization was calculated as described below.

Volume of crystallizer Vc = 100 mL

Working Pressure P_w = 120 bar = 118.43 atm

Depressurization pressure P_d = 1 atm

Volume of CO_2 in 1 atm, V_{atm} was obtained from eq 1.

Depressurization rate (mL/min) = R_d

Time required for complete depressurization (t) in sec was obtained from eq 2.

Crystal length measured in mm = l

Crystal length in meter $l_c = \frac{l}{1000}$

Growth rate R in m.s-1 was obtained from eq 3.

$$V_{atm} = \frac{P_w V_c}{P_d} \qquad (1)$$

$$t = \frac{V_{atm}}{R_d} x60 \qquad (2)$$

$$R = \frac{l_c}{t} \qquad (3)$$

For example, Growth rate of anthracene ((Table 2, in present work) in $m.s^{-1}$

$$R_{anthracene} = \frac{l_c}{t} = \frac{l_c R_d}{V_{atm}.60} = \frac{l_c R_d P_d}{P_w V_c.60000} = \frac{2x10x1}{118.43x100x60000} = 2.8 \text{ x}10^{-8}$$

Table S1: Recycle test of anthracene crystallization.[a)]

Recycle Nos.	W_0 (g)	W_c (g)	W_p (g)	W_a (g)	% Yield [(W_c/W_0) x 100]
1	2	1.35	0.52	1.48	67.09
2	2	1.30	0.54	1.46	65.0
3	2	1.33	0.43	--	66.5

a) CO_2 120 bar; temperature 45 °C; depressurization 10 mLper min. W_0 weight taken; W_c weight of crystals obtained (g) W_p weight of powder after cycle.

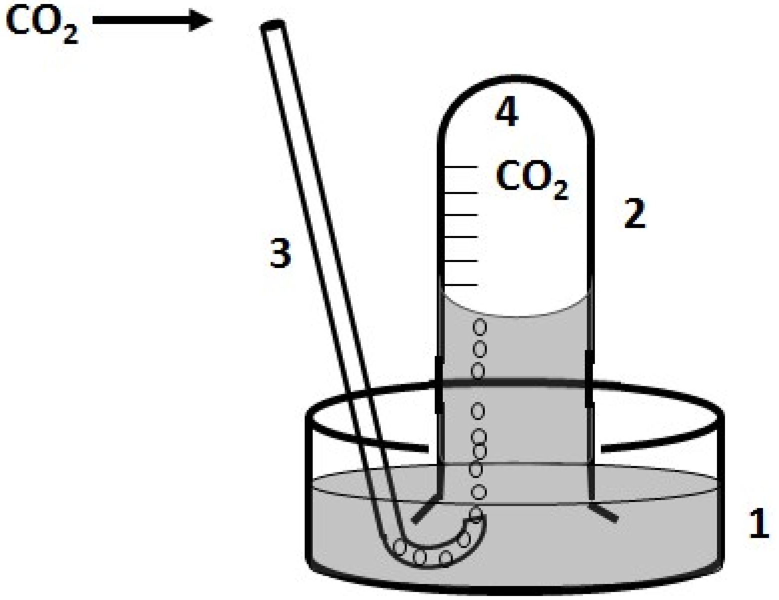

Fig S1. Pneumatic trough gas collection for measurement of depressurized CO_2 gas collected to crystallizer vent.

(a) (b)

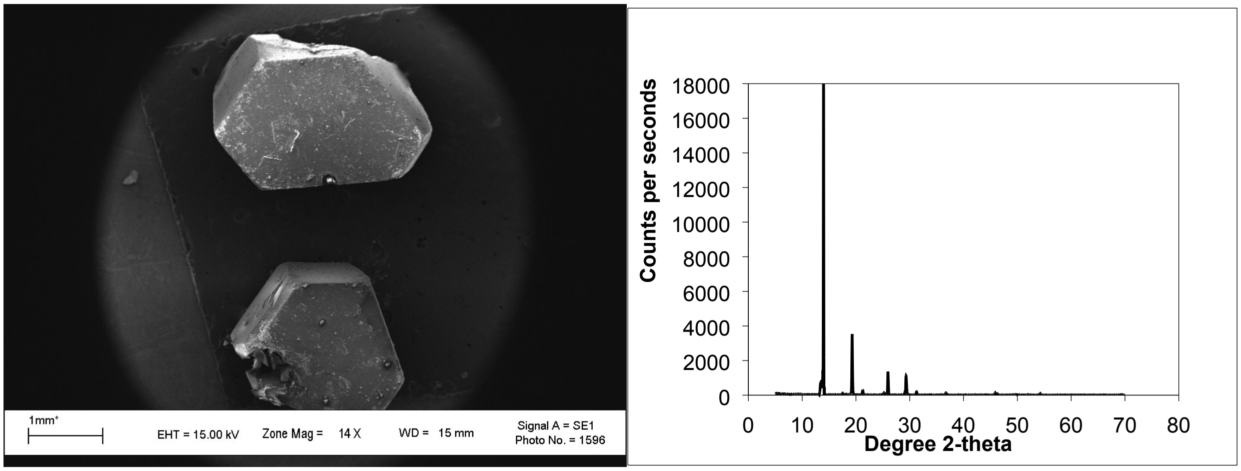

Fig. S2. a) SEM Image, b) XRD of naphthalene crystal. CO_2 120 bar, temp. 45°C, depressurization 10 mL per min, FWHM for 2θ 13.96° [001] 0.0856.

(a) (b)

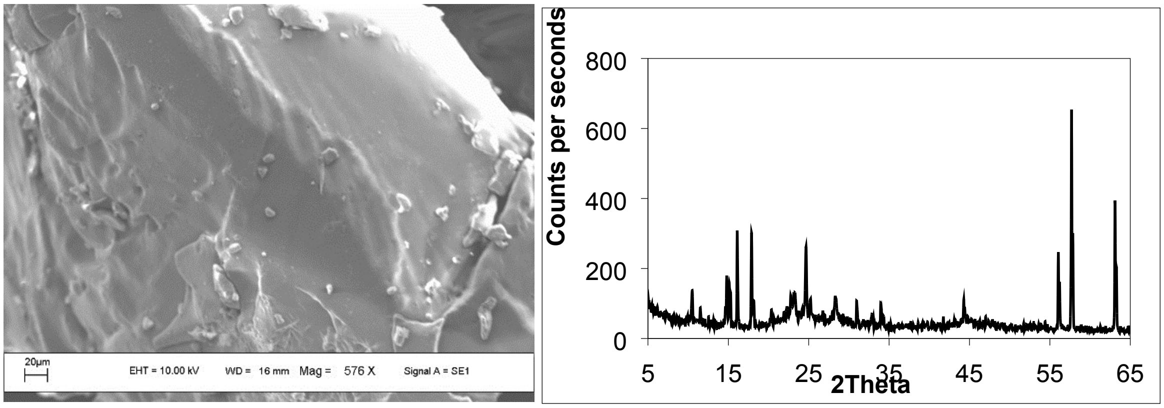

Fig. S3. a) SEM Image, b) XRD of anthracene crystal. CO_2 120 bar, temp. 45°C, depressurization rate 50 mL per min.
